# Cinnamyl 8-meth­oxy-2-oxo-2*H*-chromene-3-carboxyl­ate

**DOI:** 10.1107/S1600536809041725

**Published:** 2009-10-23

**Authors:** Cui-Lian Xu, Shan-Yu Liu, Cai-Xia Wang, Ming-Qin Zhao

**Affiliations:** a College of Sciences, Henan Agricultural University, Zhengzhou 450002, People’s Republic of China; b College of Tobacco Science, Henan Agricultural University, Zhengzhou 450002, People’s Republic of China

## Abstract

In the crystal structure of the title compound, C_20_H_16_O_5_, the mol­ecule assumes an *E* configuration with the benzene ring and chromenecarboxyl group located on opposite ends of the C=C double bond. The chromene ring system and benzene ring are oriented at a dihedral angle of 74.66 (12)°. Weak inter­molecular C—H⋯O hydrogen bonding is present in the crystal structure.

## Related literature

For applications of coumarins and related compounds, see: Hoult & Paya (1996 ▶); Yu *et al.* (2003 ▶, 2007 ▶); Finn *et al.* (2004 ▶).
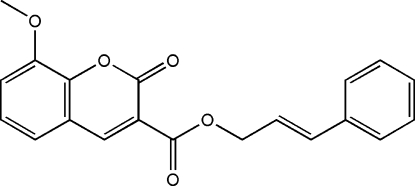


## Experimental

### 

#### Crystal data

C_20_H_16_O_5_
*M* *_r_* = 336.33
Monoclinic, 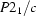

*a* = 19.226 (4) Å
*b* = 9.5483 (19) Å
*c* = 9.0046 (18) Å
β = 90.97 (3)°
*V* = 1652.8 (6) Å^3^
*Z* = 4
Mo *K*α radiation
μ = 0.10 mm^−1^
*T* = 296 K
0.20 × 0.17 × 0.17 mm

#### Data collection

Bruker SMART CCD area detector diffractometer
Absorption correction: none
4903 measured reflections
2834 independent reflections
2157 reflections with *I* > 2σ(*I*)
*R*_int_ = 0.053

#### Refinement

*R*[*F* ^2^ > 2σ(*F* ^2^)] = 0.065
*wR*(*F* ^2^) = 0.155
*S* = 1.15
2834 reflections
228 parameters
H-atom parameters constrained
Δρ_max_ = 0.24 e Å^−3^
Δρ_min_ = −0.20 e Å^−3^

### 

Data collection: *SMART* (Bruker, 2007 ▶); cell refinement: *SAINT* (Bruker, 2007 ▶); data reduction: *SAINT*; program(s) used to solve structure: *SHELXTL* (Sheldrick, 2008 ▶); program(s) used to refine structure: *SHELXTL*; molecular graphics: *SHELXTL*; software used to prepare material for publication: *SHELXTL*.

## Supplementary Material

Crystal structure: contains datablocks global, I. DOI: 10.1107/S1600536809041725/xu2613sup1.cif

Structure factors: contains datablocks I. DOI: 10.1107/S1600536809041725/xu2613Isup2.hkl

Additional supplementary materials:  crystallographic information; 3D view; checkCIF report

## Figures and Tables

**Table 1:** Hydrogen-bond geometry (Å, °)

*D*—H⋯*A*	*D*—H	H⋯*A*	*D*⋯*A*	*D*—H⋯*A*
C4—H4*A*⋯O3^i^	0.93	2.51	3.429 (3)	170
C17—H17*A*⋯O4^ii^	0.93	2.44	3.294 (4)	153

Symmetry codes: (i) 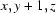; (ii) 
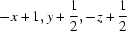
.

**Fig. 1.**
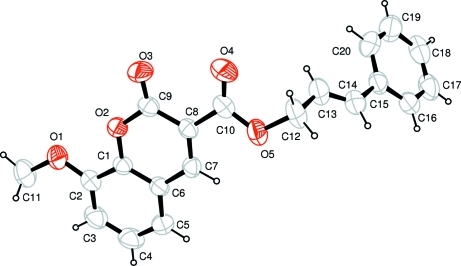
The molecular structure of the title compound. Displacement ellipsoids are drawn at the 50% probability level for non-H atoms.

## supplementary crystallographic information

### Comment

Coumarins and related compounds, kinds of plant-derived compounds, have diverse biological activities, including anti-HIV, anti-bacterial, anti-inflammatory, anti-proliferative and antioxidant properties (Hoult & Paya, 1996; Yu *et al.*, 2003; Finn *et al.*, 2004; Yu *et al.*, 2007). It thus appeared of interest to synthesize the compounds with coumarin-skeleton. As part of work, we have synthesized the title compound (I) and report its crystal structure here.

The title molecule crystallizes in the E conformation, with an C12-C13-C14-C15 torsion angle of -179.5 (3)°. The 8-methoxy-2*H*-chromen-2-one ring and the C15-benzene ring make a dihedral of 74.66 (12)°.

In the crystal structure, an intramolecular C—H···O hydrogen bond is observed and helps to stablize the conformation of the molecule.

### Experimental

A solution of cinnamic alcohol (7.2 mmol) dissolved in dried methyl dichloride (DCM) (25 ml) was added dropwise to a solution of 2-oxo-2*H*-chromene-3-acyl chloride (7.2 mmol) dissolved in DCM (25 ml) and triethylamine (1 ml) at room temperature. The reaction mixture was stirred for 24 h, monitored by TLC. The reaction mixture was neutralized with 5% HCl and washed by saturated NaHCO_3_ solution and brine, respectively. The organic phase is dried over Na_2_SO_4_ and evaporated under the reduced pressure. The resulting residue was purified by column chromatography (EtOAc:petroleum ether) to give the purified compound.

### Refinement

All H atoms were positioned geometrically and refined as riding with C—H = 0.93 (aromatic), 0.97 (methylene) and 0.96 Å (methyl), U_iso_(H) = 1.5U_eq_(C) for methyl H atoms and 1.2U_eq_(C) for the others.

### Crystal data

**Table:**

C_20_H_16_O_5_	*F*(000) = 704
*M**_r_* = 336.33	*D*_x_ = 1.352 Mg m^−^^3^
Monoclinic, *P*2_1_/*c*	Mo *K*α radiation, λ = 0.71073 Å
Hall symbol: -P 2ybc	Cell parameters from 2834 reflections
*a* = 19.226 (4) Å	θ = 3.1–24.2°
*b* = 9.5483 (19) Å	µ = 0.10 mm^−^^1^
*c* = 9.0046 (18) Å	*T* = 296 K
β = 90.97 (3)°	Block, colorless
*V* = 1652.8 (6) Å^3^	0.20 × 0.17 × 0.17 mm
*Z* = 4	

### Data collection

**Table:**

Bruker SMART CCD area detector diffractometer	2157 reflections with *I* > 2σ(*I*)
Radiation source: fine-focus sealed tube	*R*_int_ = 0.053
graphite	θ_max_ = 25.0°, θ_min_ = 1.1°
ω scans	*h* = −22→22
4903 measured reflections	*k* = −11→11
2834 independent reflections	*l* = 0→10

### Refinement

**Table:**

Refinement on *F*^2^	Secondary atom site location: difference Fourier map
Least-squares matrix: full	Hydrogen site location: inferred from neighbouring sites
*R*[*F*^2^ > 2σ(*F*^2^)] = 0.065	H-atom parameters constrained
*wR*(*F*^2^) = 0.155	*w* = 1/[σ^2^(*F*_o_^2^) + (0.0659*P*)^2^ + 0.2012*P*] where *P* = (*F*_o_^2^ + 2*F*_c_^2^)/3
*S* = 1.15	(Δ/σ)_max_ = 0.001
2834 reflections	Δρ_max_ = 0.24 e Å^−^^3^
228 parameters	Δρ_min_ = −0.20 e Å^−^^3^
0 restraints	Extinction correction: *SHELXL*, Fc^*^=kFc[1+0.001xFc^2^λ^3^/sin(2θ)]^-1/4^
Primary atom site location: structure-invariant direct methods	Extinction coefficient: 0.024 (3)

### Special details

**Table:**

Geometry. All esds (except the esd in the dihedral angle between two l.s. planes) are estimated using the full covariance matrix. The cell esds are taken into account individually in the estimation of esds in distances, angles and torsion angles; correlations between esds in cell parameters are only used when they are defined by crystal symmetry. An approximate (isotropic) treatment of cell esds is used for estimating esds involving l.s. planes.
Refinement. Refinement of *F*^2^ against ALL reflections. The weighted *R*-factor *wR* and goodness of fit *S* are based on *F*^2^, conventional *R*-factors *R* are based on *F*, with *F* set to zero for negative *F*^2^. The threshold expression of *F*^2^ > σ(*F*^2^) is used only for calculating *R*-factors(gt) *etc*. and is not relevant to the choice of reflections for refinement. *R*-factors based on *F*^2^ are statistically about twice as large as those based on *F*, and *R*- factors based on ALL data will be even larger.

### Fractional atomic coordinates and isotropic or equivalent isotropic displacement parameters (Å^2^)

**Table:**

	*x*	*y*	*z*	*U*_iso_*/*U*_eq_	
O1	0.04001 (9)	0.88854 (18)	0.70962 (19)	0.0610 (5)	
O2	0.11713 (7)	0.72996 (15)	0.54027 (17)	0.0463 (4)	
O3	0.15130 (10)	0.51497 (17)	0.4995 (2)	0.0705 (6)	
O4	0.25790 (11)	0.4732 (2)	0.3000 (3)	0.0885 (7)	
O5	0.28952 (8)	0.66346 (19)	0.17300 (19)	0.0617 (5)	
C1	0.12199 (11)	0.8722 (2)	0.5210 (2)	0.0414 (6)	
C2	0.08113 (11)	0.9568 (3)	0.6115 (3)	0.0497 (6)	
C3	0.08597 (14)	1.0996 (3)	0.5946 (3)	0.0652 (8)	
H3A	0.0593	1.1578	0.6536	0.078*	
C4	0.13001 (15)	1.1585 (3)	0.4910 (3)	0.0710 (8)	
H4A	0.1323	1.2554	0.4816	0.085*	
C5	0.17013 (13)	1.0757 (3)	0.4023 (3)	0.0605 (7)	
H5A	0.1992	1.1160	0.3328	0.073*	
C6	0.16698 (11)	0.9293 (2)	0.4174 (3)	0.0445 (6)	
C7	0.20856 (11)	0.8339 (2)	0.3351 (2)	0.0450 (6)	
H7A	0.2383	0.8693	0.2638	0.054*	
C8	0.20641 (10)	0.6940 (2)	0.3568 (2)	0.0429 (6)	
C9	0.15868 (11)	0.6355 (2)	0.4665 (3)	0.0460 (6)	
C10	0.25260 (12)	0.5959 (3)	0.2770 (3)	0.0525 (6)	
C11	−0.00432 (15)	0.9739 (3)	0.7994 (3)	0.0743 (8)	
H11A	−0.0314	0.9150	0.8626	0.112*	
H11B	−0.0349	1.0278	0.7362	0.112*	
H11C	0.0237	1.0360	0.8592	0.112*	
C12	0.33877 (13)	0.5785 (3)	0.0894 (3)	0.0697 (8)	
H12A	0.3470	0.6224	−0.0059	0.084*	
H12B	0.3189	0.4867	0.0709	0.084*	
C13	0.40569 (13)	0.5629 (3)	0.1714 (3)	0.0646 (7)	
H13A	0.4053	0.5142	0.2608	0.078*	
C14	0.46490 (14)	0.6123 (3)	0.1279 (3)	0.0639 (7)	
H14A	0.4640	0.6613	0.0386	0.077*	
C15	0.53343 (12)	0.6000 (3)	0.2031 (3)	0.0560 (7)	
C16	0.58725 (14)	0.6863 (3)	0.1617 (4)	0.0741 (9)	
H16A	0.5804	0.7481	0.0830	0.089*	
C17	0.65113 (15)	0.6830 (3)	0.2345 (5)	0.0831 (10)	
H17A	0.6861	0.7445	0.2067	0.100*	
C18	0.66314 (14)	0.5891 (3)	0.3481 (4)	0.0741 (9)	
H18A	0.7059	0.5874	0.3980	0.089*	
C19	0.61138 (15)	0.4981 (4)	0.3866 (3)	0.0763 (9)	
H19A	0.6195	0.4320	0.4608	0.092*	
C20	0.54716 (14)	0.5041 (3)	0.3158 (3)	0.0694 (8)	
H20A	0.5123	0.4426	0.3443	0.083*	

### Atomic displacement parameters (Å^2^)

**Table:**

	*U*^11^	*U*^22^	*U*^33^	*U*^12^	*U*^13^	*U*^23^
O1	0.0600 (10)	0.0639 (11)	0.0597 (11)	0.0079 (9)	0.0171 (8)	−0.0059 (9)
O2	0.0487 (9)	0.0410 (9)	0.0494 (10)	0.0015 (7)	0.0053 (7)	−0.0008 (7)
O3	0.0869 (14)	0.0375 (10)	0.0877 (14)	0.0016 (9)	0.0227 (10)	0.0041 (9)
O4	0.1028 (16)	0.0562 (13)	0.1075 (17)	0.0296 (11)	0.0322 (13)	0.0052 (11)
O5	0.0522 (10)	0.0719 (12)	0.0615 (12)	0.0113 (9)	0.0125 (8)	−0.0069 (9)
C1	0.0391 (12)	0.0364 (12)	0.0486 (14)	0.0004 (10)	−0.0026 (10)	−0.0021 (10)
C2	0.0444 (13)	0.0505 (15)	0.0541 (15)	0.0053 (11)	−0.0019 (11)	−0.0051 (12)
C3	0.0668 (17)	0.0523 (16)	0.0767 (19)	0.0122 (13)	0.0061 (14)	−0.0112 (14)
C4	0.0784 (19)	0.0382 (14)	0.096 (2)	0.0072 (13)	0.0005 (17)	−0.0028 (15)
C5	0.0590 (16)	0.0446 (14)	0.0783 (19)	−0.0029 (12)	0.0079 (13)	0.0063 (13)
C6	0.0394 (12)	0.0392 (12)	0.0550 (15)	0.0021 (10)	−0.0010 (10)	−0.0012 (11)
C7	0.0370 (12)	0.0503 (14)	0.0477 (14)	−0.0026 (10)	0.0007 (9)	0.0011 (11)
C8	0.0364 (12)	0.0444 (13)	0.0476 (14)	0.0026 (10)	−0.0039 (10)	−0.0026 (10)
C9	0.0472 (13)	0.0401 (14)	0.0506 (15)	0.0028 (10)	−0.0024 (10)	−0.0044 (11)
C10	0.0470 (14)	0.0573 (16)	0.0531 (16)	0.0102 (12)	−0.0031 (11)	−0.0058 (13)
C11	0.0676 (18)	0.090 (2)	0.0663 (19)	0.0167 (16)	0.0152 (14)	−0.0173 (16)
C12	0.0520 (16)	0.093 (2)	0.0642 (18)	0.0203 (14)	0.0082 (13)	−0.0162 (16)
C13	0.0517 (15)	0.0826 (19)	0.0598 (17)	0.0113 (14)	0.0072 (12)	−0.0058 (15)
C14	0.0605 (16)	0.0681 (17)	0.0635 (17)	0.0112 (14)	0.0127 (13)	−0.0002 (14)
C15	0.0484 (14)	0.0513 (14)	0.0690 (18)	0.0042 (12)	0.0139 (12)	−0.0074 (13)
C16	0.0617 (18)	0.0521 (16)	0.109 (2)	0.0106 (14)	0.0295 (16)	0.0079 (16)
C17	0.0542 (18)	0.0535 (17)	0.143 (3)	−0.0068 (14)	0.0329 (18)	−0.012 (2)
C18	0.0490 (16)	0.078 (2)	0.096 (2)	0.0006 (15)	0.0078 (15)	−0.0310 (19)
C19	0.0669 (18)	0.091 (2)	0.071 (2)	0.0004 (17)	0.0080 (15)	0.0048 (16)
C20	0.0549 (16)	0.0741 (19)	0.080 (2)	−0.0136 (14)	0.0122 (14)	0.0070 (16)

### Geometric parameters (Å, °)

**Table:**

O1—C2	1.362 (3)	C11—H11A	0.9600
O1—C11	1.438 (3)	C11—H11B	0.9600
O2—C1	1.373 (3)	C11—H11C	0.9600
O2—C9	1.382 (3)	C12—C13	1.480 (4)
O3—C9	1.198 (3)	C12—H12A	0.9700
O4—C10	1.194 (3)	C12—H12B	0.9700
O5—C10	1.349 (3)	C13—C14	1.299 (4)
O5—C12	1.465 (3)	C13—H13A	0.9300
C1—C6	1.394 (3)	C14—C15	1.476 (4)
C1—C2	1.398 (3)	C14—H14A	0.9300
C2—C3	1.375 (4)	C15—C16	1.379 (3)
C3—C4	1.389 (4)	C15—C20	1.389 (4)
C3—H3A	0.9300	C16—C17	1.383 (4)
C4—C5	1.370 (4)	C16—H16A	0.9300
C4—H4A	0.9300	C17—C18	1.377 (4)
C5—C6	1.405 (3)	C17—H17A	0.9300
C5—H5A	0.9300	C18—C19	1.370 (4)
C6—C7	1.428 (3)	C18—H18A	0.9300
C7—C8	1.351 (3)	C19—C20	1.381 (4)
C7—H7A	0.9300	C19—H19A	0.9300
C8—C9	1.469 (3)	C20—H20A	0.9300
C8—C10	1.485 (3)		
			
C2—O1—C11	116.7 (2)	H11A—C11—H11B	109.5
C1—O2—C9	122.93 (18)	O1—C11—H11C	109.5
C10—O5—C12	116.4 (2)	H11A—C11—H11C	109.5
O2—C1—C6	121.0 (2)	H11B—C11—H11C	109.5
O2—C1—C2	117.3 (2)	O5—C12—C13	111.3 (2)
C6—C1—C2	121.7 (2)	O5—C12—H12A	109.4
O1—C2—C3	126.0 (2)	C13—C12—H12A	109.4
O1—C2—C1	116.1 (2)	O5—C12—H12B	109.4
C3—C2—C1	117.9 (2)	C13—C12—H12B	109.4
C2—C3—C4	121.3 (3)	H12A—C12—H12B	108.0
C2—C3—H3A	119.4	C14—C13—C12	124.9 (3)
C4—C3—H3A	119.4	C14—C13—H13A	117.6
C5—C4—C3	120.9 (2)	C12—C13—H13A	117.6
C5—C4—H4A	119.6	C13—C14—C15	127.8 (3)
C3—C4—H4A	119.6	C13—C14—H14A	116.1
C4—C5—C6	119.5 (2)	C15—C14—H14A	116.1
C4—C5—H5A	120.3	C16—C15—C20	117.2 (3)
C6—C5—H5A	120.3	C16—C15—C14	119.8 (3)
C1—C6—C5	118.8 (2)	C20—C15—C14	123.0 (2)
C1—C6—C7	117.2 (2)	C15—C16—C17	121.5 (3)
C5—C6—C7	124.0 (2)	C15—C16—H16A	119.2
C8—C7—C6	122.5 (2)	C17—C16—H16A	119.2
C8—C7—H7A	118.7	C18—C17—C16	120.2 (3)
C6—C7—H7A	118.7	C18—C17—H17A	119.9
C7—C8—C9	119.6 (2)	C16—C17—H17A	119.9
C7—C8—C10	122.2 (2)	C19—C18—C17	119.2 (3)
C9—C8—C10	118.1 (2)	C19—C18—H18A	120.4
O3—C9—O2	115.8 (2)	C17—C18—H18A	120.4
O3—C9—C8	127.6 (2)	C18—C19—C20	120.3 (3)
O2—C9—C8	116.62 (19)	C18—C19—H19A	119.9
O4—C10—O5	123.1 (2)	C20—C19—H19A	119.9
O4—C10—C8	125.8 (3)	C19—C20—C15	121.5 (3)
O5—C10—C8	111.2 (2)	C19—C20—H20A	119.3
O1—C11—H11A	109.5	C15—C20—H20A	119.3
O1—C11—H11B	109.5		
			
C9—O2—C1—C6	4.0 (3)	C7—C8—C9—O3	−179.1 (2)
C9—O2—C1—C2	−174.71 (19)	C10—C8—C9—O3	−1.1 (3)
C11—O1—C2—C3	2.6 (4)	C7—C8—C9—O2	1.1 (3)
C11—O1—C2—C1	−177.7 (2)	C10—C8—C9—O2	179.11 (18)
O2—C1—C2—O1	−0.4 (3)	C12—O5—C10—O4	−1.3 (3)
C6—C1—C2—O1	−179.15 (19)	C12—O5—C10—C8	178.20 (18)
O2—C1—C2—C3	179.3 (2)	C7—C8—C10—O4	172.9 (3)
C6—C1—C2—C3	0.6 (3)	C9—C8—C10—O4	−5.0 (4)
O1—C2—C3—C4	179.8 (2)	C7—C8—C10—O5	−6.5 (3)
C1—C2—C3—C4	0.0 (4)	C9—C8—C10—O5	175.55 (18)
C2—C3—C4—C5	−0.1 (4)	C10—O5—C12—C13	−83.6 (3)
C3—C4—C5—C6	−0.5 (4)	O5—C12—C13—C14	−115.1 (3)
O2—C1—C6—C5	−179.8 (2)	C12—C13—C14—C15	−179.5 (3)
C2—C1—C6—C5	−1.2 (3)	C13—C14—C15—C16	−164.1 (3)
O2—C1—C6—C7	−1.3 (3)	C13—C14—C15—C20	15.9 (4)
C2—C1—C6—C7	177.37 (19)	C20—C15—C16—C17	−3.4 (4)
C4—C5—C6—C1	1.1 (4)	C14—C15—C16—C17	176.6 (3)
C4—C5—C6—C7	−177.4 (2)	C15—C16—C17—C18	2.2 (4)
C1—C6—C7—C8	−1.3 (3)	C16—C17—C18—C19	0.7 (4)
C5—C6—C7—C8	177.1 (2)	C17—C18—C19—C20	−2.3 (4)
C6—C7—C8—C9	1.4 (3)	C18—C19—C20—C15	0.9 (4)
C6—C7—C8—C10	−176.50 (19)	C16—C15—C20—C19	1.9 (4)
C1—O2—C9—O3	176.3 (2)	C14—C15—C20—C19	−178.2 (3)
C1—O2—C9—C8	−3.8 (3)		

### Hydrogen-bond geometry (Å, °)

**Table:**

*D*—H···*A*	*D*—H	H···*A*	*D*···*A*	*D*—H···*A*
C4—H4A···O3^i^	0.93	2.51	3.429 (3)	170
C17—H17A···O4^ii^	0.93	2.44	3.294 (4)	153

Symmetry codes: (i) *x*, *y*+1, *z*; (ii) −*x*+1, *y*+1/2, −*z*+1/2.
